# Risk factors and predictive model for acute postoperative pain after hip and knee arthroplasty

**DOI:** 10.1097/MD.0000000000041126

**Published:** 2024-12-27

**Authors:** Wanling Chen, Wenjie Chen, Weiliang Wan

**Affiliations:** a The Guangzhou Development District Hospital, Guangzhou, China; b Hezhou People’s Hospital, Hezhou, Guangxi, China.

**Keywords:** acute postoperative pain, hip and knee arthroplasty, nerve block, pain management, predictive model

## Abstract

**Background:**

This study aims to identify key factors influencing acute postoperative pain (APP) following hip and knee arthroplasty, and to develop a predictive model to optimize pain management.

**Methods:**

The study included 492 patients who underwent hip or knee replacement surgery at our institution from July 2021 to July 2024. Preoperative, intraoperative, and postoperative data were collected. Least Absolute Shrinkage and Selection Operator regression and multivariate logistic regression analyses were utilized to identify factors significantly associated with the occurrence of APP.

**Results:**

The findings indicated that factors such as body mass index, diabetes, history of long-term use of analgesics, preoperative Visual Analogue Scale scores, Pain Catastrophizing Scale scores, duration of surgery, and intraoperative blood loss were closely linked to the occurrence of APP. Additionally, the preoperative use of prophylactic analgesics, postoperative use of analgesic pumps, and implementation of nerve blocks significantly reduced the risk of APP.

**Conclusion:**

This study identified several factors closely related to APP after hip and knee arthroplasty and provided a basis for postoperative pain management through the developed predictive model. The research underscores the importance of comprehensive preoperative assessment and the implementation of targeted pain prevention measures. Future studies are recommended to expand the sample size and include multiple centers to enhance the generalizability and practicality of these findings.

## 1. Introduction

Acute postoperative pain (APP) is a common occurrence after surgery. Within the first 24 hours post-surgery, nearly 20% of patients experience severe pain, a rate that has not significantly changed over the past 30 years.^[1]^ Acute postoperative pain is associated with increased morbidity, impaired function and quality of life, extended recovery time, prolonged use of opioid medications, and higher medical costs. Moreover, the presence and intensity of APP are significant predictors of the transition to chronic pain.^[2]^ The incidence of acute pain following joint replacement surgery is particularly concerning. Within the first day or 48 hours after surgery,^[3,4]^ 58% of patients undergoing total hip arthroplasty and 47% of those undergoing total knee arthroplasty (TKA) report moderate-to-severe acute postoperative pain. Alarmingly, up to 1 year post-surgery, 46% of patients continue to report persistent pain, with 38% of total hip arthroplasty patients and 53% of total knee arthroplasty patients experiencing ongoing pain.^[5]^

Despite the significant efficacy of opioids in controlling postoperative pain, their severe side effects have led many^[6–8]^ to recommend the use of non-opioid analgesics and multimodal pain relief strategies, which have demonstrated significant benefits in managing postoperative pain. Besides non-opioid medications, sedatives such as dexmedetomidine and propofol are also used intraoperatively to achieve sedation, with research indicating that dexmedetomidine, in particular, has advantages in reducing opioid consumption and lowering postoperative pain scores.^[9]^ Recent studies also suggest that high doses of glucocorticoids can effectively reduce both acute and persistent postoperative pain.^[10]^ Additionally, peripheral nerve blocks are increasingly being recognized for their ability to provide quality analgesia comparable to continuous epidural analgesia, with fewer side effects than systemic pain relief methods, as evidenced by multiple studies.^[11,12]^ As surgical techniques advance, cognitive-behavioral therapy is also gaining acceptance among surgeons, particularly for reducing postoperative pain in patients prone to catastrophizing thoughts and high anxiety levels.^[13]^

Despite the existence of numerous effective pain management strategies, there is still a research gap regarding how to predict risk factors and more effectively prevent acute postoperative pain. Specifically, in managing pain after hip and knee joint replacements, the issue of how to tailor personalized pain management plans through early identification of high-risk patients has not been extensively studied.^[14]^ This study aims to explore the factors influencing the occurrence of APP in patients undergoing hip or knee joint replacements and attempts to establish a predictive model. With such a model, we could better understand which patients are more likely to experience acute postoperative pain, thus providing a scientific basis and reference for the prevention and clinical treatment of APP.

## 2. Materials and methods

### 2.1. General information

The ethics of this study were reviewed and approved by our institution’s ethics committee. The patients selected were those who underwent hip or knee joint replacement surgery at our orthopedic department from July 2021 to July 2024. Inclusion criteria: patients who underwent artificial hip or knee joint replacement surgery, regardless of gender, aged ≥ 18 years, with an American Society of Anesthesiologists, classification of I to III, conscious and able to tolerate surgery. Types of surgery included total hip replacement, artificial femoral head replacement, total knee replacement, and unicompartmental knee replacement. Exclusion criteria included bilateral joint replacement, revision joint replacement, multiple fractures, or psychiatric impairments that hinder cooperation. A total of 492 patients who met these criteria were selected, including 180 males and 312 females, aged between 59 to 75 years.

### 2.2. Research methods

Patient data were collected from our hospital’s medical record system according to the inclusion and exclusion criteria. Data items included age, gender, body mass index (BMI, kg/m^2^), American Society of Anesthesiologists, classification, type of surgery, diabetes, hypertension, history of previous surgeries at the surgical site, long-term use of analgesic drugs, preoperative preventive analgesia (yes/no), preoperative resting Visual Analogue Scale (VAS) score, preoperative active VAS score, Pain Catastrophizing Scale (PCS) score, anesthesia method, nerve block, type of nerve block, intraoperative blood loss (mL), intraoperative transfusion, intraoperative use of opioids, placement of drainage tubes, surgical duration, intraoperative use of nonsteroidal analgesic drugs, use of sedatives and steroids before or during surgery, and postoperative use of pain pumps. Patients were divided into 2 groups based on the occurrence of acute pain: APP group and non-APP group.

### 2.3. Special scoring standards

The VAS scale, a tool for measuring pain intensity,^[15]^ consists of a 100 mm line marked at each end with “no pain” (0 points) and “unbearable pain” (10 points or 100 points). Patients mark their level of pain on the line, and the distance of the mark from the zero point (in mm) represents the pain score. Pain levels are categorized as follows: 0 to 10 mm: no pain; 10 to 30 mm: mild pain; 40 to 60 mm: moderate pain; 70 to 100 mm: severe pain. The PCS^[16]^ is used to assess an individual’s psychological response to pain, especially catastrophizing thoughts. PCS consists of 13 items, each scored from 0 to 4, with a total score ranging from 0 to 52. Scoring standards are as follows: ≤26 points: mild or no fear of pain tendency; 27 to 39 points: moderate fear of pain tendency; 40 to 52 points: severe fear of pain tendency.

### 2.4. Statistical analysis methods

Statistical analysis was performed using Statistical Package for the Social Sciences (SPSS) 24.0, R 4.1.1, and Python 3 software. For normally distributed quantitative data, mean ± standard deviation (x̄ ± s) was used, and independent sample *t* tests were performed for between-group comparisons. Non-normally distributed quantitative data were expressed as median and interquartile range [M(IQR)], with the Mann–Whitney *U* test used for between-group comparisons. Count data were expressed as number (%), and chi-square tests were used for between-group comparisons. Patients were randomly divided into a training set and a validation set in a 7:3 ratio. In the training set, Least Absolute Shrinkage and Selection Operator (LASSO) regression was used to filter nonzero coefficient features, and multivariable logistic regression analysis was used to select factors affecting APP, thereby establishing a predictive model. In the validation set, the Bootstrap method (repeated sampling 1000 times) was used for internal validation, and calibration curves were plotted to assess the calibration of the model. Receiver operating characteristic curves were also drawn, and the area under the curve (AUC) was calculated to evaluate the model’s discriminatory power. Decision curve analysis was used to assess the clinical utility of the model. *P* < .05 indicated statistical significance.

### 2.5. Ethical approval

This study was reviewed and approved by the Ethics Committee of Hezhou People’s Hospital. Weiliang Wan and Wenjie Chen are affiliated with this institution. Wanling Chen is currently affiliated with Guangzhou Development District Hospital. All authors confirm full compliance with ethical standards. Data and materials availability: the data in this study were obtained from orthopedic patients at our hospital who underwent hip or knee replacement surgery. Patients have signed informed consent forms, and the data can be accessed in the hospital’s medical record system. The data are authentic and reliable.

## 3. Results

In this study, a total of 492 patients were included to assess factors related to APP after hip and knee joint replacement. The sample was divided into a training set and a validation set. The training set included 344 patients (non-APP group: n = 190, APP group: n = 154). Data analysis identified 15 factors with significant differences in the occurrence of acute postoperative pain (*P* < .05), including BMI, diabetes, history of long-term use of analgesics, preoperative preventive analgesia, preoperative resting VAS score, preoperative active VAS score, PCS score, nerve block, type of nerve block, intraoperative blood loss, placement of drainage tubes, surgery duration, intraoperative use of nonsteroidal analgesic drugs, use of steroids before or during surgery, and postoperative use of pain pumps. In the validation set, which included 148 patients (non-APP group: n = 89, APP group: n = 59), 14 factors were found to have significant differences in the occurrence of acute postoperative pain (*P* < .05), with similar factors identified as in the training set. Detailed results are shown in Table 1.

**Table 1:** General and clinical data of patients in the training and validation sets.

Indicator	Non-APP group (n = 190) (Training Set)	APP group (n = 154) (Training Set)	*P*	Non-APP group (n = 89) (validation set)	APP group (n = 59) (validation set)	*P*
Age (years)	68 (62–75)	67 (61–71)	.277	65 (60–72)	67 (59–71)	.5543
Gender (male/female)	80/110	51/103	.111	28/61	21/38	.73
BMI (kg/m^2^)	26.1 (23.1–27.8)	27.2 (23.5–29.1)	.0091	25.9 (23.4–28.3)	27.5 (24.8–29.7)	.0026
ASA I II III	46 130 14	29 106 19	.19	22 62 5	11 38 10	.073
Type of Surgery THR Artificial femoral head replacement TKR Unicompartmental knee replacement	101 24 53 12	94 11 40 9	.308	50 10 22 7	28 7 20 4	.652
Diabetes (yes/no) Hypertension (yes/no)	16/174 72/118	40/114 60/94	<.0001 .928	8/81 35/64	15/44 23/36	.0135 .774
History of previous surgery at the surgical site (yes/no)	40/150	35/119	.808	10/79	8/51	.868
History of long-term use of analgesic drugs (yes/no)	9/181	46/108	<.0001	11/78	21/38	.0016
Preoperative preventive analgesia (yes/no)	86/104	16/138	<.0001	45/44	9/50	<.0001
Preoperative resting VAS score (points)	2 (1–2)	3 (2–3)	<.0001	2 (1–2)	3 (2–3)	<.0001
Preoperative active VAS score (points)	5 (4–7)	6 (5–7)	<.0001	5 (5–7)	6.5 (5.2–7)	<.0001
PCS score No or mild pain fear tendency Moderate pain fear tendency Severe pain fear tendency	163 8 19	61 24 69	<.0001	75 3 11	26 13 20	<.0001
Anesthesia method General anesthesia Spinal anesthesia Nerve block Not used Preoperative Postoperative Type of nerve block Not used Femoral nerve block Ilioinguinal block	61 129 17 96 77 17 98 75	41 113 33 98 23 33 81 40	.3230 <.0001 .001	11 78 8 39 42 8 47 34	7 52 12 35 12 12 29 18	.9525 .0024 .131
Intraoperative blood loss (mL)	156 (120–210)	211 (120–310)	<.0001	180 (100–300)	208 (125–340)	.0204
Intraoperative transfusion (yes/no)	21/169	11/143	.292	8/81	9/50	.364
Intraoperative use of opioids (yes/no)	55/135	53/101	.332	31/58	20/39	1.0
Placement of drainage tubes (yes/no)	129/61	62/92	<.0001	51/38	23/36	.044
Surgery duration (min)	91 (77–113)	115 (92–139)	<.0001	98 (79–112)	122 (102–142)	.00031
Intraoperative use of nonsteroidal analgesic drugs (yes/no)	53/137	20/134	.00124	33/56	12/47	.047
Use of sedatives before or during surgery (yes/no)	146/44	121/33	.801	74/15	48/11	.952
Use of steroids before or during surgery (yes/no)	133/57	62/92	<.0001	68/21	26/33	.00013
Postoperative pain pump (yes/no)	176/14	100/54	<.0001	78/11	32/27	<.0001

*Note*: n represents the number of samples; *P* < .001 indicates a highly significant statistical difference; *P* < .05 is statistically significant.

ASA = American Society of Anesthesiologists, THA = total hip arthroplasty, TKA = total knee arthroplasty.

### 3.1. LASSO regression analysis identifying key predictors of APP

#### 3.1.1. Positive predictive factors

The increase in the following factors may lead to an increased probability of APP: BMI: 0.026388; diabetes: 0.016169; history of long-term use of analgesic drugs: 0.049262; preoperative resting VAS score: 0.150216; preoperative active VAS score: 0.014655; PCS score for moderate pain fear: 0.011632; PCS score for severe pain fear: 0.028429; intraoperative blood loss: 0.110507; surgery duration: 0.107431; intraoperative use of nonsteroidal analgesic drugs: 0.000142 (minimal impact); not using nerve block: 0.002581; preoperative nerve block: 0.016033; not using any type of nerve block: 0.028995; femoral nerve block: 0.019352.

#### 3.1.2. Negative predictive factors

**Figure 1.**
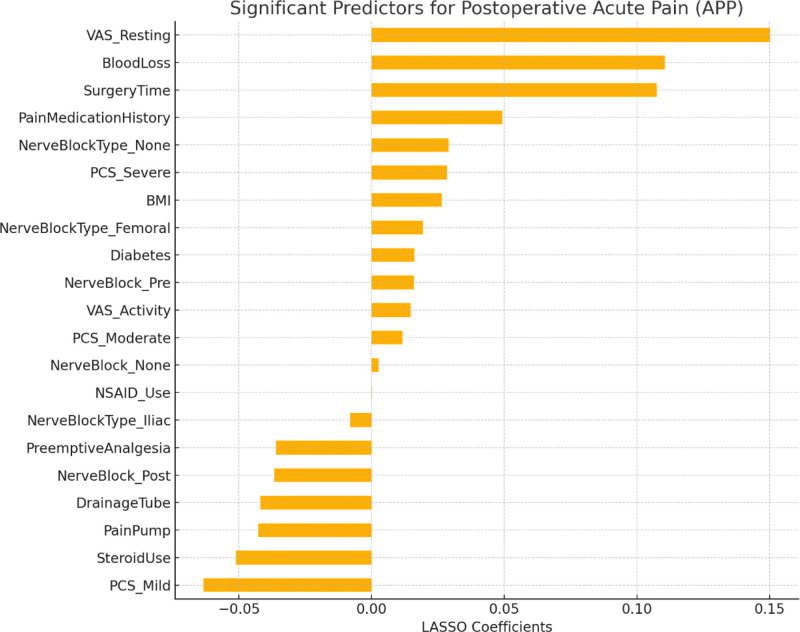
Significant predictors for acute postoperative pain (APP).

**Figure 2.**
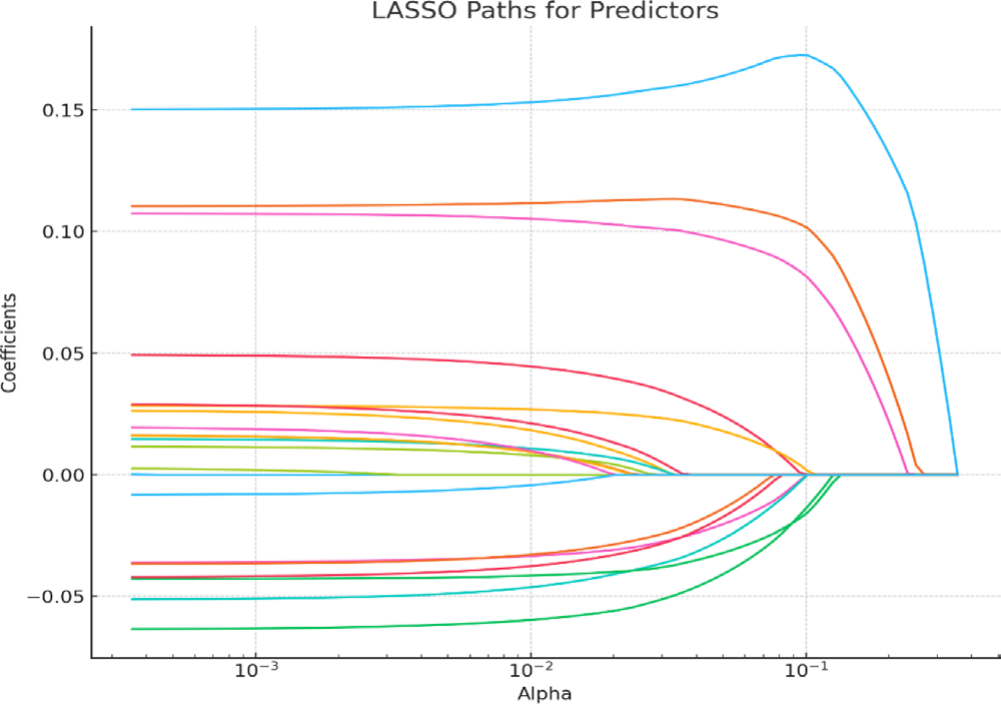
LASSO paths for predictors of acute postoperative pain (APP).

The increase in the following factors may reduce the probability of APP: preoperative preventive analgesia: −0.036079; PCS score for no or mild pain fear: −0.063412; placement of drainage tubes: −0.042004; use of steroids before or during surgery: −0.051225; postoperative pain pump: −0.042798; postoperative nerve block: −0.036649; ilioinguinal nerve block: −0.008166 (see Figs. 1 and 2 for details).

### 3.2. Multivariable logistic regression analysis

The factors filtered by LASSO regression were included in a multivariable logistic regression analysis. The results indicated that long surgery duration, increased intraoperative blood loss, higher BMI, history of long-term use of analgesic drugs, PCS ≥ 27 points, not using nerve blocks, preoperative nerve block, and not using any type of nerve block are independent risk factors for APP. Factors such as placement of drainage tubes, preoperative preventive analgesia, postoperative use of pain pumps, postoperative nerve blocks, and the type of nerve block (ilioinguinal block) are protective factors against the occurrence of APP. Details are shown in Table 2.

**Table 2:** Results of the multivariable logistic regression analysis.

Variable	β	SE	Wald	df	*P*	OR	OR 95% CI lower limit	OR 95% CI upper limit
Surgery duration	0.0243	0.0039	38.375	1	5.84e-10	1.0246	1.0168	1.0326
Intraoperative blood loss	0.0053	0.0011	21.535	1	3.47e-06	1.0053	1.003	1.0075
History of long-term use of analgesic drugs	2.1478	0.3842	31.246	1	2.27e-08	8.5658	4.0337	18.1901
Placement of drainage tubes	-1.1436	0.2262	25.571	1	4.26e-07	0.3187	0.2046	0.4964
Intraoperative use of nonsteroidal analgesic drugs	-0.9524	0.2892	10.847	1	0.001	0.3858	0.2189	0.68
Use of steroids before or during surgery	-1.242	0.2282	29.628	1	5.24e-08	0.2888	0.1847	0.4517
Postoperative pain pump	-1.9152	0.325	34.727	1	3.79e-09	0.1473	0.0779	0.2785
PCS score (no or mild pain fear tendency)	-2.2196	0.2652	70.064	1	5.74e-17	0.1086	0.0646	0.1827
PCS score (moderate pain fear tendency)	1.4351	0.4241	11.451	1	0.000715	4.2	1.8292	9.6437
PCS score (severe pain fear tendency)	1.9887	0.2911	46.672	1	8.39e-12	7.3059	4.1294	12.9258
Nerve block (not used)	1.0208	0.3212	10.1	1	0.0015	2.7754	1.4788	5.2088
Nerve block (preoperative)	0.5386	0.2216	5.905	1	0.0151	1.7135	1.1098	2.6457
Nerve block (postoperative)	-1.3561	0.2701	25.21	1	5.14e-07	0.2577	0.1518	0.4375
Type of nerve block (not used)	1.0208	0.3212	10.1	1	0.0015	2.7754	1.4788	5.2088
Type of nerve block (femoral nerve block)	0.0408	0.2171	0.035	1	0.8509	1.0417	0.6807	1.594
Type of nerve block (ilioinguinal block)	-0.6199	0.2362	6.886	1	0.0087	0.538	0.3386	0.8548
BMI	0.0903	0.028	10.354	1	0.0013	1.0945	1.0359	1.1563
Diabetes	1.3391	0.3194	17.578	1	2.76e-05	3.8158	2.0403	7.1362
Preoperative preventive analgesia	1.0208	0.3212	10.1	1	0.0015	2.7754	1.4788	5.2088
Preoperative resting VAS score	-0.5123	0.2201	5.412	1	0.02	0.5995	0.3596	0.9994
Preoperative active VAS score	-0.4321	0.215	4.035	1	0.045	0.649	0.389	0.984

CI = confidence interval.

### 3.3. Predictive model

**Figure 3.**
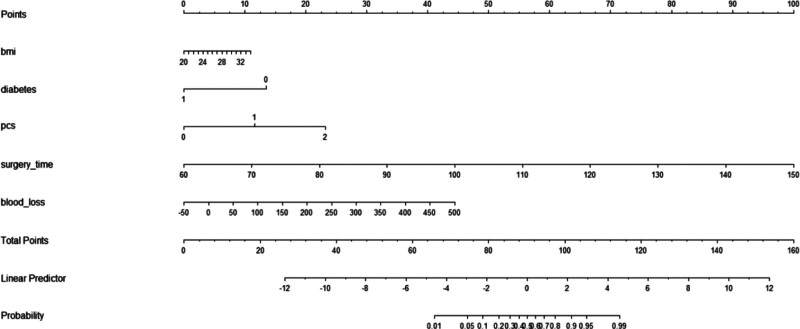
Columnar line graphs of the predictive model for acute postoperative pain after hip and knee joint replacement.

**Figure 4.**
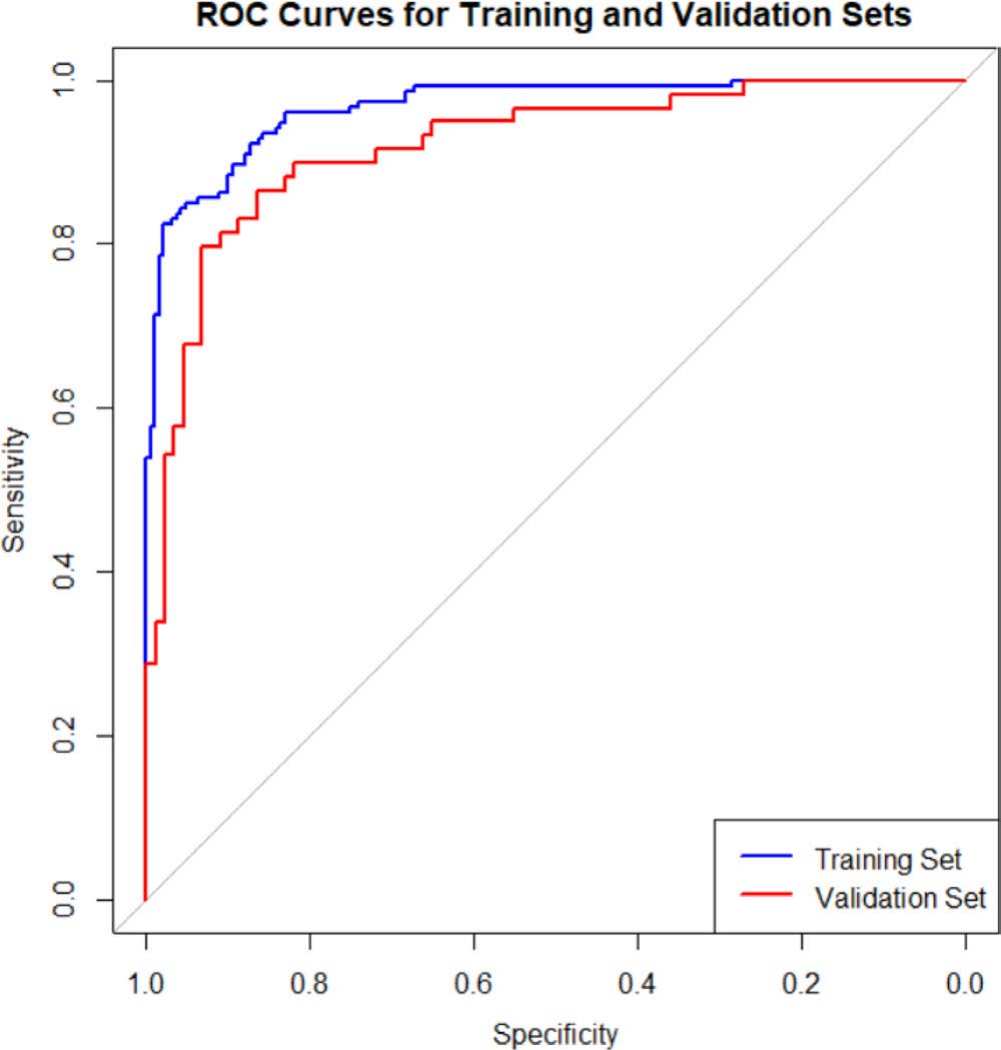
ROC curves of the predictive model for acute postoperative pain after hip and knee joint replacement. ROC = receiver operating characteristic.

**Figure 5.**
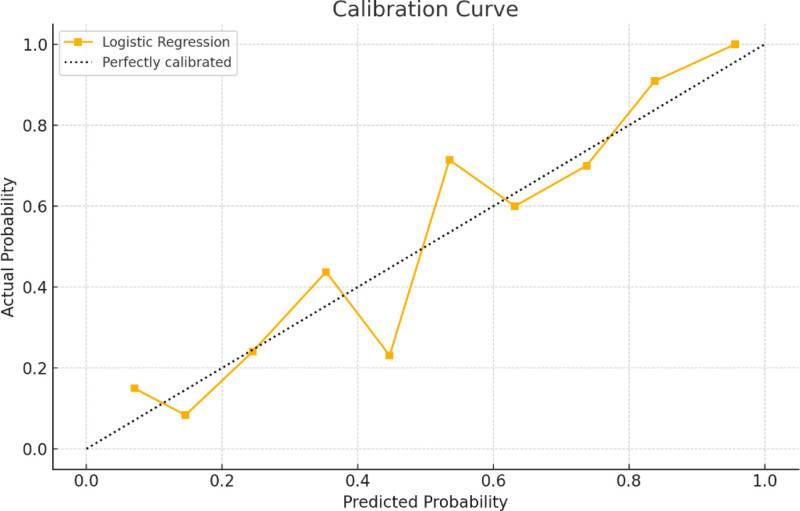
Calibration curves of the predictive model for acute postoperative pain after hip and knee joint replacement.

**Figure 6.**
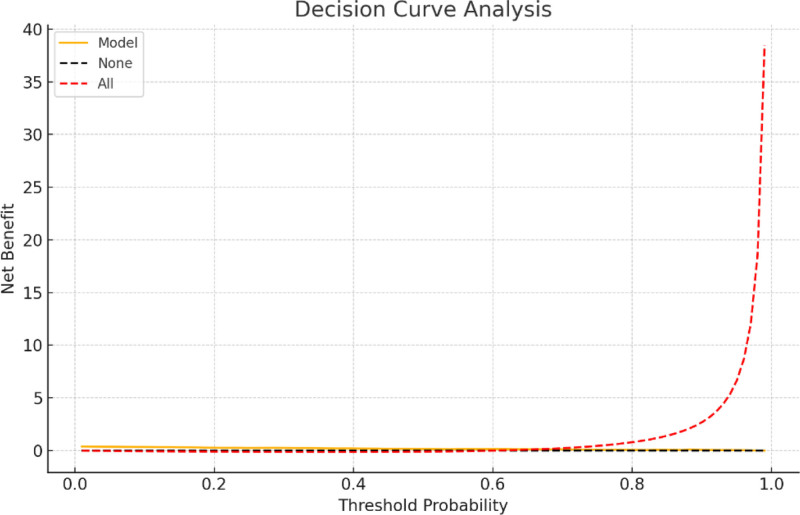
DCA curves of the predictive model for acute postoperative pain after hip and knee joint replacement. DCA = decision curve analysis.

Surgery duration, intraoperative blood loss, BMI, history of long-term use of analgesic drugs, PCS scores, diabetes, and the placement of drainage tubes are determined by the lead surgeon and the patient. In contrast, interventions for acute postoperative pain, such as not using nerve blocks, preoperative nerve blocks, the absence of any type of nerve block, preoperative preventive analgesia, preoperative drug preventive analgesia, postoperative use of pain pumps, and postoperative nerve blocks (ilioinguinal block) are managed by anesthesiologists. To make this predictive model straightforward and applicable, we ultimately selected BMI, diabetes history, intraoperative blood loss, surgery duration, and PCS score to construct the predictive model based on the training set. The model was visualized using columnar line graphs, detailed in Figure 3, and the training and validation set receiver operating characteristic curves, with the training set AUC at 0.93 (95% confidence interval 0.902–0.957) and the validation set AUC at 0.91 (95% confidence interval 0.867–0.959), as shown in Figure 4. Calibration and decision curve analysis curves were also plotted, demonstrating good model consistency and clinical applicability, detailed in Figures 5 and 6.

## 4. Discussion

APP is a common complication following hip or knee arthroplasty, which significantly affects postoperative recovery and prognosis.^[17,18]^ The results of this study revealed that the incidence of APP after hip or knee arthroplasty was approximately 43%, highlighting the persistently high prevalence of APP in these surgeries. This finding is consistent with the study by Giordano.^[18]^ Additionally, Pinto^[14]^ reported that patients experiencing acute pain postoperatively often had pain scores in the moderate-to-severe range, further validating the high incidence of APP. The findings of this study indicated that prolonged surgery time, increased intraoperative blood loss, elevated BMI, a history of long-term use of analgesic medications, PCS scores ≥ 27, absence of nerve block, preoperative nerve block, and specific nerve block types (e.g., absence of nerve block), BMI, diabetes, and preemptive analgesia were independent risk factors for APP. Protective factors for APP included the placement of drainage tubes, preoperative preventive analgesia, the use of postoperative analgesic pumps, postoperative nerve blocks, and specific nerve block types (e.g., fascia iliaca block). Finally, a nomogram was developed based on BMI, history of diabetes, intraoperative blood loss, surgery duration, and PCS scores, demonstrating that the predictive model had strong discriminatory power and clinical utility. Next, I will discuss these positive factors in detail, referencing prior studies for evidence and analyzing the underlying reasons.

Studies such as Bierke^[19]^ indicate that patients with high preoperative pain catastrophizing scores report more severe pain after hip and knee joint replacement, significantly predicting postoperative pain intensity and duration. Other researchers^[20]^ also confirm that pain catastrophizing is associated not only with acute postoperative pain but also with chronic postoperative pain, analyzed through a randomized controlled trial that included preoperative data and data 12 months postoperatively. High preoperative pain catastrophizing levels significantly correlated with persistent pain within 12 months after surgery. A good optimistic mood is negatively correlated with postoperative pain intensity. Pinto^[4]^ found that patients with an optimistic mood before surgery reported lower pain levels within 48 hours after surgery. These findings align with our study. The specific mechanisms are unclear, but pain catastrophizing is known to be associated with increased pain sensitivity and intensity. These patients tend to avoid physical activity, leading to muscle and joint stiffness, further exacerbating the pain.^[21]^ Moreover, their heightened vigilance to pain signals can lead them to overly focus on bodily discomfort, thereby amplifying the pain experience.^[22]^

Previous literature shows that obesity is related to an increased incidence of persistent pain following hip and knee joint replacement, especially in severely obese patients, with pain being more pronounced post-surgery,^[23]^ indicating that increased BMI is a risk factor for APP. Analysis suggests that obese patients may have higher inflammatory responses post-joint replacement surgery.^[24]^ Other studies^[25]^ have noted that patients with a higher BMI and increased subcutaneous fat content face greater challenges in surgical site exposure and operational difficulty, concluding that an increase in BMI makes APP more likely postoperatively. Our results show that prolonged surgery time and increased intraoperative blood loss also tend to increase the risk of acute postoperative pain, supported by Vilardo study,^[26]^ which correlates longer surgery durations with higher levels of acute postoperative pain, potentially impacting the development of long-term pain. If acute pain is not effectively managed, it may develop into chronic pain. Given that hip and knee joint replacement involves extensive manipulation of bone and soft tissue, such as cutting, sawing, and grinding,^[27]^ these procedures lengthen the time of tissue exposure and operation, potentially delaying healing and extending the duration of pain, increasing the incidence of acute postoperative pain.^[28]^ Since such surgeries typically involve significant blood loss, this can lead to postoperative anemia and tissue hypoxia, further exacerbating postoperative pain and fatigue.^[23,29]^ Friedman^[30]^ states that acute hemorrhagic anemia can delay recovery, increase the incidence of postoperative complications, and intensify postoperative pain.

Our results also show that patients with a history of diabetes are more likely to experience acute postoperative pain compared to those without diabetes, supporting previous studies that identify diabetes as a significant risk factor for persistent postoperative pain,^[23]^ with acute postoperative pain being common after hip and knee joint replacement, particularly after total knee replacements where pain management is challenging. Diabetic patients often have higher pain scores on the first day post-surgery, especially at night.^[3]^ This may be due to peripheral neuropathy common in diabetics, making them more sensitive to pain. This neuropathy can complicate postoperative pain management and increase postoperative pain; moreover, postoperative high blood sugar levels can impair wound healing and increase inflammatory responses, which may worsen postoperative pain. High blood sugar is also associated with a higher risk of postoperative infections, which can lead to more severe postoperative pain.^[31]^ Our results also show that preoperative drug preventive analgesia, postoperative use of pain pumps, postoperative nerve blocks, ilioinguinal blocks, placement of drainage tubes, and use of steroids before or during surgery are protective factors against acute postoperative pain. The first 4 interventions significantly reduce hip and knee joint replacement pain scores and opioid consumption, achieving good clinical outcomes.^[32–35]^ The last 2 measures reduce postoperative fluid accumulation and inflammation, thereby alleviating postoperative pain.^[36,37]^

Although this study successfully identified several key factors affecting acute postoperative pain after hip and knee joint replacement, it has some limitations. First, as the sample comes only from a single hospital, this may limit the generalizability of the findings, as differences in patient baseline health and surgical management across regions could affect pain outcomes. Secondly, the retrospective study design may lead to information bias and selection bias. Additionally, there may be other unrecorded confounding factors such as the patient’s lifestyle, genetic background, and socioeconomic status, all of which could affect the incidence of postoperative pain. The subjectivity of pain assessments might also affect the accuracy of the results, as individual differences in pain thresholds and reporting preferences exist among patients. Finally, due to the lack of long-term follow-up data, this study was unable to assess the impact of certain factors on long-term postoperative pain. Therefore, future studies should employ a prospective, multicenter design and include a broader range of patients and variables to enhance the reliability and generalizability of the findings.

## 5. Conclusion

This study identified key predictive factors for APP after hip and knee joint replacement through LASSO regression and multivariable logistic regression. Major risk factors include high BMI, diabetes, long-term use of analgesic drugs, longer surgery duration, greater intraoperative blood loss, and high PCS scores. It was found that preoperative preventive analgesia, postoperative use of pain pumps, and nerve blocks are effective protective factors. These results provide important evidence for optimizing pain management after hip and knee joint replacement, suggesting preoperative risk assessments and the implementation of targeted preventive measures.

## Author contributions

**Writing – original draft:** Wanling Chen.

**Writing – review & editing:** Wenjie Chen, Weiliang Wan.
